# Efficacy and safety of immune checkpoint inhibitors-combined antiangiogenic drugs in the treatment of hepatocellular carcinoma: A systematic review and meta analysis

**DOI:** 10.3389/fonc.2022.964779

**Published:** 2022-08-18

**Authors:** Yu Zhong, Hong Huo, Shuqi Dai, Su Li

**Affiliations:** ^1^ Department of Pharmacy, Cancer Hospital of China Medical University, Liaoning Cancer Hospital & Institute, Shenyang, China; ^2^ Department of Pharmaceutics, School of Pharmacy, China Medical University, Shenyang, China

**Keywords:** immune checkpoint inhibitors, antiangiogenic drug, combination therapy, hepatocellular carcinoma, tumor treatment

## Abstract

**Background:**

Hepatocellular carcinoma is a pathological type of liver cancer and accounts for the majority of primary liver cancers. We conducted a meta-analysis to evaluate the efficacy and safety of immune checkpoint inhibitors in combination with antiangiogenic drugs in the treatment of hepatocellular carcinoma.

**Methods:**

We searched scientific literature databases and clinical trials databases through May 2022 for required studies. Progression-free survival was taken as the main outcome, and overall survival, response rate and adverse events as secondary outcomes. These data were extracted, combined and used for meta-analysis to compare the treatment effect and safety of immune checkpoint inhibitors combined with antiangiogenic drugs in patients with advanced/unresectable/metastatic hepatocellular carcinoma.

**Results:**

This study included 3 randomized controlled trials and 6 single-arm trials of immune checkpoint inhibitors in combination with antiangiogenic drugs in hepatocellular carcinoma. Meta-analysis showed that compared with single use, combination of the two can significantly improve PFS (HR=5.93, 95% CI=5.41, 6.45) and OS (HR=15.84, 95% CI=15.39, 16.28). The ORR and DOR of patients with combination therapy were HR=19.11, 95% CI=15.99, 22.22 and HR=12.26, 95% CI=10.32, 14.21, respectively. Common adverse reactions to combination therapy included hypertension (26.8%), diarrhea (23.6%), fatigue (23.8%), decreased appetite (22.8%), hypothyroidism (9.9%), and rash (14.5%).

**Conclusion:**

In the treatment of advanced/unresectable/metastatic hepatocellular carcinoma, immune checkpoint inhibitors combined with antiangiogenic drugs achieved better survival benefits than alone. In addition, the combination therapy has tolerable safety.

## Introduction

Primary liver cancer is a malignant tumor of the digestive system with high incidence worldwide, and most patients are already in the advanced stage of cancer when they are found to have liver cancer. According to the new data released by GLOBOCAN 2020, the annual number of new cases of liver cancer in the world reached 906,000, ranking sixth among malignant tumors and 830,000 deaths, ranking third among malignant tumors (1). The main pathiogyical type of primary liver cancer is hepatocellular carcinoma (HCC), accounting for 85%-90% (2). HCC occurs in the liver which is severely damaged by chronic injury or inflammation (3–5).

Immune checkpoint inhibitors (ICIs) are new types of monoclonal antibodies. It works by inhibiting the function of inhibitory immune receptors and by stimulating the immune system’s antitumor response (6). Cancer immunotherapy against antibodies against programmed cell death-1 (PD-1)/programmed cell death ligand 1 (PD-L1) axis shows excellent effect in the treatment of liver cancer (7). Immune checkpoint inhibitors of PD-1/PD-L1 are important anti-tumor immunotherapy drugs, representing a major breakthrough in the treatment of advanced HCC. Tumor immunotherapy with PD-1 blockers shows a good effect in the treatment of liver cancer. The factors that affect the clinical outcome of PD-1 inhibitors include specific receptors, signal pathways and inflammatory genes. The findings of these factors suggest that researchers can use combination therapy to reduce the impact of other factors on the treatment effect of PD-1/PD-L1 inhibitors (8). At present, the National Drug Administration (NMPA) in China has approved the PD-1 antibodies for HCC indications: Camrelizumab, Tislelizumab, Sintilimab; and the PD-L1 antibody for HCC indications: Atezolizumab. PD-1 antibodies for FDA approval of HCC indications: Nivolumab, Pembrolizumab; PD-L1 antibodies for approval of HCC indications: Atezolizumab. The effect of immunologic drug monotherapy for unresectable HCC is unsatisfactory, and the ORR of PD-1 monotherapy for HCC is 17%-20%. But so far, the survival superiority of monotherapy with ICIs including PD-1 has not been demonstrated in randomized studies (9).

Tumor angiogenesis is a complex process because multiple signaling pathways are involved. Among them, vascular endothelial growth factor (VEGF)/VEGF receptor 2 (VEGFR2) signal pathway is one of the important pathways of tumor angiogenesis and plays an important role in regulating immune response. By disrupting blood vessel supply and starving tumors of nutrients and oxygen, antiangiogenic drugs are also a promising treatment. This is primarily achieved by blocking the VEGF/VEGF receptor VEGFR signaling pathway that is active in the tumor microenvironment under hypoxic conditions (10). Therefore, inhibition of this pathway can promote vascular normalization, increase lymphocyte infiltration in tumor, and attenuates the function of inhibitory immune cell phenotype. Currently, the global standard first-line systemic regimen for unresectable or metastatic HCC is VEGFR tyrosine kinase inhibitors. Although these drugs have a certain degree of survival benefit, they are accompanied by considerable toxicity. According to RECIST1.1, the objective response rate (ORR) of lenvatinib and sorafenib for unresectable HCC was 19% and 7%, respectively (9).

Various types of immune cells are present in the liver, and they produce different cytokines and growth factors in response to local stimuli. Thus, these immune cells establish an immune microenvironment to maintain a balance between immune tolerance and hepatic immune activation (11). The high efficacy of combination therapy with ICIs and antiangiogenic drugs is not only due to their additive effects on tumor growth, but also because both focus on targeting the tumor microenvironment, reprogramming the immunosuppressive microenvironment into an immunostimulatory one. Among them, the reason why VEGF inhibitors can reprogram the immunosuppressive tumor microenvironment into an immunostimulatory environment is that such drugs can increase the antigen presentation of dendritic cells, promote the activation of T cells in the priming phase, and improve T cells. Migration from lymph nodes to tumor sites. Furthermore, these drugs inhibit the generation of Tregs, TAMs and MDSCs at tumor sites and negatively regulate the expression of immunosuppressive cytokines. Therefore, the combination of ICIs and antiangiogenic agent exhibits a potential synergistic anti-tumor effect (12, 13). At present, there are a number of clinical trials to verify whether the addition of antiangiogenic drugs can improve the efficacy of immune checkpoint inhibitors in tumor treatment. And when used in the comprehensive treatment of liver cancer, impressive anti-tumor effects have been observed (14–16). For example, the median survival time involved in the natural progression of disease in patients with advanced HCC is about 8 months, while the combination of anti-PD-L1 antibodies-atrazumab and anti-VEGF antibodies-bevacizumab more than doubled this life expectancy and improved patient reported outcome indicators (17).

Although the combination therapy of ICIs and antiangiogenic drugs has shown stronger antitumor activity in hepatocellular carcinoma, the clinical trials of the related combination have been reported in small population sizes and with specific series of Adverse events are not fully defined, including evidence of increased risk of gastrointestinal, cutaneous, and vascular events. Importantly, there has been no systematic attempt to synthesize data on the efficacy and safety of combination therapy with these drugs, and considering that the combination therapy of ICIs and antiangiogenic drugs offers new hope in the treatment of HCC, we believe that it is crucial to clarify the efficacy and safety of these drug combinations for cancer treatment. Therefore, we conducted this systematic review and meta-analysis after searching extensive literature to analyze the treatment efficacy and safety of ICIs-combined antiangiogenic drugs in advanced/unresectable/metastatic HCC.

## Materials and methods

### Search strategy

We have carried out a systematic literature search in electronic databases (Pubmed, Web of Science, Cochrane Library), and the final search time is up to May 2022. The retrieval is carried out by the combination of subject headings and free words, and adjusted according to the characteristics of each database. The search strategy mainly includes three parts: (1) Words related to ‘immune checkpoint inhibitors’: (ie ‘immune checkpoint inhibitors’, ‘PD-1 inhibitors’, ‘PD-L1 inhibitors’, ‘nivolumab’, ‘pembrolizumab’, ‘sintilimab’, ‘camrelizumab’, ‘toripalimab’, ‘tislelizumab’, ‘atezolizumab’, ‘durvalumab’, ‘avelumab’). (2) Words related to ‘liver cancer’: (ie ‘Liver cancer’, ‘Hepatocellular carcinoma’). (3) The search filter is set to ‘Clinical Trial’.

### Inclusion and exclusion criteria

Potential trials were screened on the basis of: (1) expected Phase I, II and III clinical trials and expanded access (i.e., external clinical trials) programs; (2) clinical investigations of patients with hepatocellular carcinoma and the fact that some participants have been assigned to PD-1/PD-L1 in combination with antiangiogenic drugs; and (3) recording adverse event-related events or efficacy comparisons.

### Data extraction and definitions

In order to understand all the baselines included in the study, we extracted the following information: the first author, the number of patients involved in the study, treatment scheme, and the basic characteristics of the participants. The main outcome is progression-free survival (PFS), the secondary outcome is overall survival (OS), objective response rate (ORR), and duration of response (DOR). The response criteria of the above extracted data are all RECIST1.1. Based on the safety results of a combination of immune checkpoint inhibitors and antiangiogenic drugs, we considered the following adverse events of grade 1-2 as clinical endpoints: hypertension, diarrhea, fatigue, decreased appetite, hypothyroidism and rash.

### Evaluation of quality

The collected RCTs and nonrandomized studies were assessed using the Jadad scoring tool (18) and the nonrandomized study methodological index (19), respectively. RCTs with scores ≥4 and nonrandomized studies with scores ≥8 were considered high-quality reports; RCTs with scores ≤3 and nonrandomized studies with scores <8 were considered low-quality reports. All included studies were assessed to have a low risk of bias (20). The results of quality evaluation are illustrated in **Table 1**. As shown in the table, 3 RCTs and 6 nonrandomized controlled studies were of high quality.

**Table 1:** Basic characteristics of studies included in this meta-analysis.

Author(issuing time)	Trial design	Patients, N	Age, years	Characteristics of hepatocellular carcinoma	Interventions		Qualityscore (Jadad/ MINORS)
					Experiment group	Control group	
Yung-Jue Bang, 2020 (21)	ST phase Ia/b	28	63 (27~87)	advanced / metastatic	Durvalumab+ Ramucirumab	None	10 (full score: 16 points)
Hoffmann-La Roche Ltd 2020 (22)	RCT phase III	558	(18~85+)	advanced / metastatic	Atezolizumab + Bevacizumab	Sorafenib	4 (full score: 7 points)
Zhenggang Ren 2020 (23)	ST Phase II	24	(18+)	unresectable / metastatic	Sintilimab + Bevacizumab biosimilar	None	9 (full score: 16 points)
	RCT phase III	571	Sintilimab–bevacizumab biosimilar group: 53 (21~82) Sorafenib group: 54 (28~77)	unresectable / metastatic	Sintilimab + Bevacizumab biosimilar	Sorafenib	5 (full score: 7 points)
Michael S Lee, 2020 (24)	ST phase Ib	104	62 (23~82)	unresectable	Atezolizumab + Bevacizumab	None	10 (full score: 16 points)
	RCT phase Ib	119	Atezolizumab plus bevacizumab (n=60): 60 (22~82); Atezolizumab monotherapy (n=59): 63 (23~85)	unresectable	Atezolizumab + Bevacizumab	Atezolizumab	5 (full score: 7 points)
Masatoshi Kudo, 2021 (25)	ST phase Ib	22	68.5 (20~84)	advanced / metastatic	Avelumab + Axitinib	None	9 (full score: 16 points)
Richard S. Finn, 2020 (26)	ST phase Ib	100	66.5 (47~86)	unresectable	Pembrolizumab + Lenvatinib	None	9 (full score: 16 points)
Jianming Xu, 2019 (27)	ST phase Ia/b	18	49 (29-64)	advanced	SHR-1210 + Apatinib	None	10 (full score: 16 points)

(RCT, randomized-controlled trial; ST, single-arm trial).

### Statistical analysis

Stata16.0 and Revman 5.4 of 64-bit Windows were used for statistical analysis. The difference between combination therapy and single treatment was estimated by combining effect size or HR (hazard ratio) and 95% confidence interval (CI). Effect sizes, HR and OR estimates were summarized using random or fixed-effects models, and heterogeneity between studies was assessed by P-value and I^2^ statistic, with a threshold of p<0.1. Homogeneous data (I^2^<50%) were pooled with a fixed-effects model, and heterogeneous data (I^2^≥50%) were pooled with a random-effects model. The symmetry of visual observations of funnel plots and the Egger’s test were used to assess publication bias.

## Result

### Study selection

**Figure 1:**
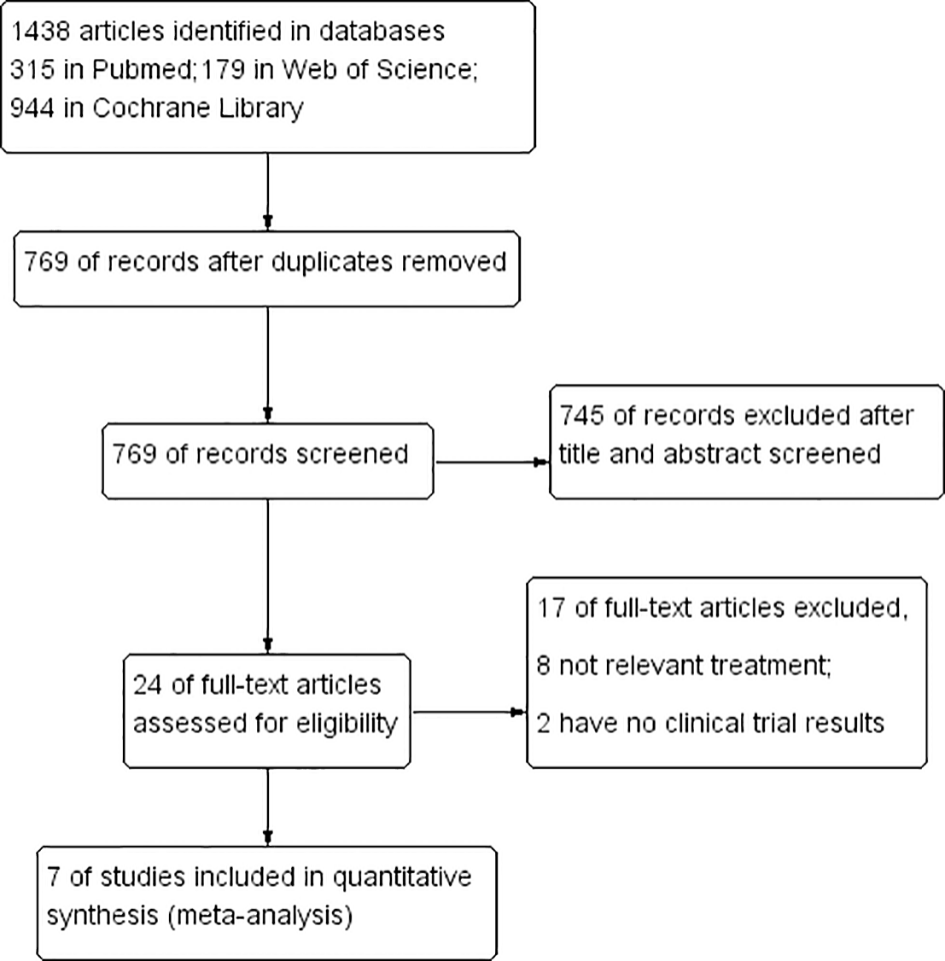
The Prisma search strategy flowchart followed in article search and selection in this study. This systematic review includes 7 studies, including 3 randomized controlled trials and 6 single-arm trials..

We searched the database for 1438 studies (including 315 in PubMed, 179 in Web of Science, and 944 in Cochrane Library). After eliminating duplicates (n=769), browse and filter the titles and abstracts. The remaining 24 studies were screened in full text, and 7 articles were finally included according to the inclusion criteria. The flow chart of the following search strategy is shown in **Figure 1**.

### Study characteristics

This systematic review includes 9 studies, including 3 randomized controlled trials and 6 single-arm trials, all published between 2019 and 2021. The clinical trial patients in the searched literature were advanced/unresectable/metastatic hepatocellular carcinoma patients. Among them, patients received PD-1/PD-L1 inhibitors including avelumab, pembrolizumab, durvalumab, atezolizumab, SHR-1210 (anti-PD-1 antibody), sintilimab and antiangiogenesis inhibitors including axitinib, lenvatinib, ramucirumab, bevacizumab, apatinib, bevacizumab biosimilar. The basic characteristics of the studies included in this meta-analysis are shown in **Table 1**.

### Publication bias test

**Figure 2:**
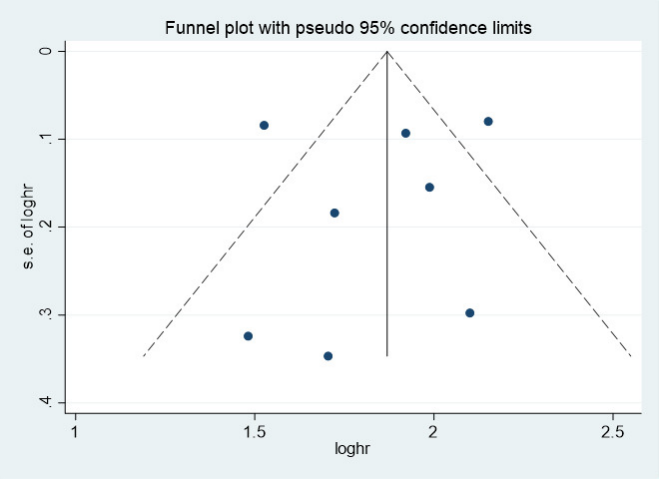
The funnel plot of the risk of bias. SE, standard error.

**Figure 3:**
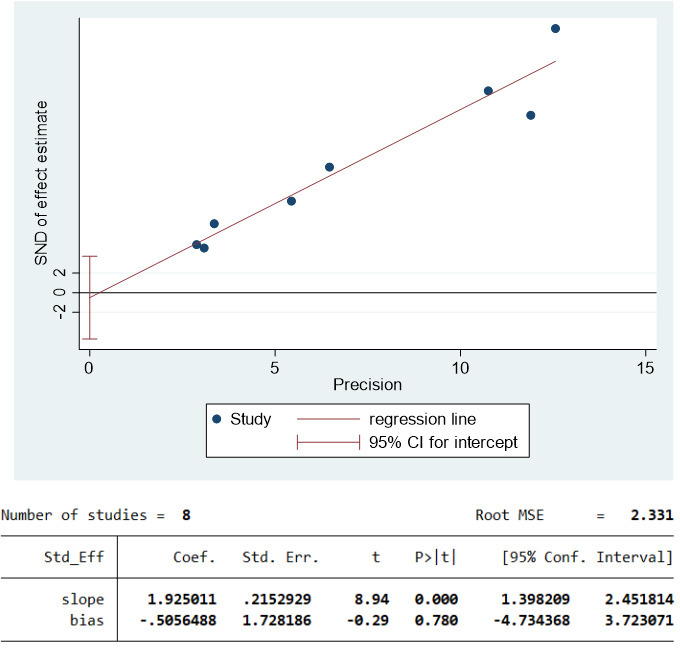
Egger’s test results.

Publication bias was assessed for 8 clinical trials in the included seven articles. The funnel plot shows that most studies are in the upper part of the ‘inverted funnel’ and fewer studies are in the base, and the left and right are basically symmetrical. Egger’s test showed that P=0.780>0.05, so the included study could not be considered to have publication bias. See **Figure 2** funnel plot and **Figure 3** Egger’s test result chart for details.

### Median progression-free survival and overall survival

Three studies (21–23) involving 610 patients with advanced/unresectable/metastatic HCC reported median OS. The OS of the included studies were HR=15.84, 95% CI=15.39, 16.28, I^2^= 68.3%, p=0.043. Clinical trials of F. Hoffmann-La Roche Ltd (22) showed that the OS of PD-1 combined with antiangiogenic drugs and without PD-1 were HR=19.22, 95% CI=17.02, 23.66 and HR=13.40, 95% CI=11.37, 16.85.

All studies involving 1,520 patients with advanced/unresectable/metastatic HCC reported median PFS. The PFS of the included studies were HR=5.93, 95% CI=5.41, 6.45, I^2^= 76.3%, p=0.000. Research by Michael S Lee et al. (24) showed that the PFS of PD-1 combined with antiangiogenic drugs and without antiangiogenic drugs were HR=5.6, 95% CI=3.6, 7.40 and HR=3.40, 95% CI=1.90, 5.20. Clinical trials of F. Hoffmann-La Roche Ltd (22) showed that the PFS of PD-1 combined with antiangiogenic drugs and without PD-1 were HR=6.83, 95% CI=5.75, 8.28 and HR=4.27, 95% CI=3.98, 5.55. Research by Zhenggang Ren et al. (23) showed that the PFS of PD-1 combined with antiangiogenic drugs and without PD-1 were HR=4.60, 95% CI=4.10, 5.70 and HR=2.80, 95% CI=2.70, 3.20.

**Figure 4:**
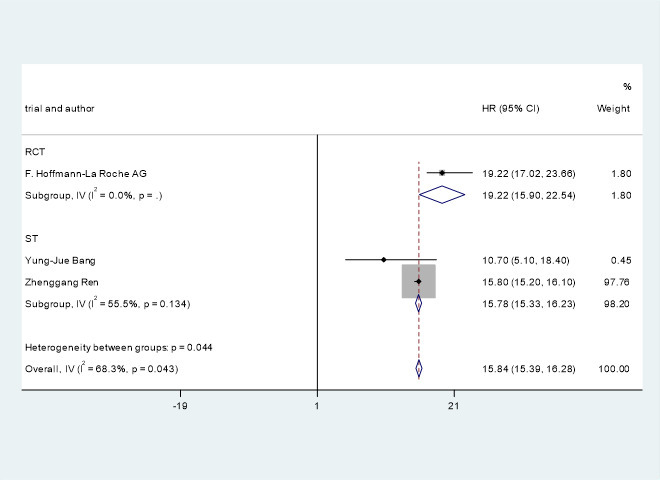
Pooled analysis of OS in patients with HCC treated with immune checkpoint inhibitors combined with antiangiogenic drugs..

**Figure 5:**
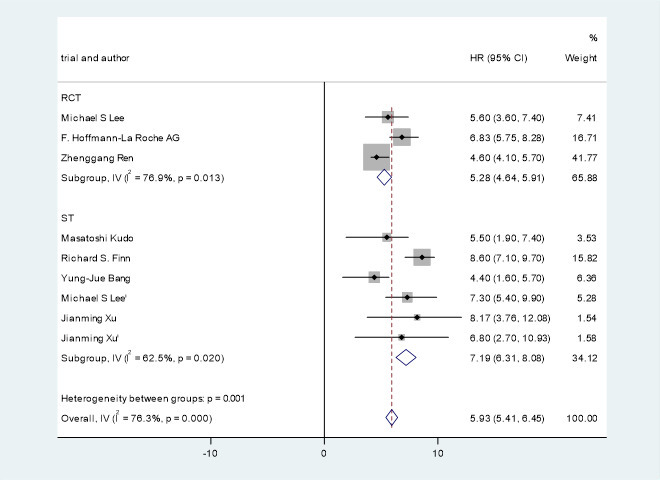
Pooled analysis of PFS in patients with HCC treated with immune checkpoint inhibitors combined with antiangiogenic drugs.

A pooled analysis of OS and PFS in HCC patients treated with immune checkpoint inhibitors combined with antiangiogenic drugs is shown in **Figures 4**, **5**.

### Response rate

The meta-analysis showed that the combined ORR (22, 25–27) and DOR (25, 27) of PD-1 combined with antiangiogenic inhibitors in hepatocellular carcinoma was HR=19.11, 95% CI=15.99, 22.22, I^2^= 92.7%, p=0.000 and HR=12.26, 95% CI=10.32, 14.21, I^2^= 95.7%, p=0.000. Clinical trials of F. Hoffmann-La Roche Ltd (22) showed that the ORR of PD-1 combined with antiangiogenic drugs and without PD-1 were HR=27.30, 95% CI=22.54, 32.48 and HR=11.90, 95% CI=7.35, 18.03.

**Figure 6:**
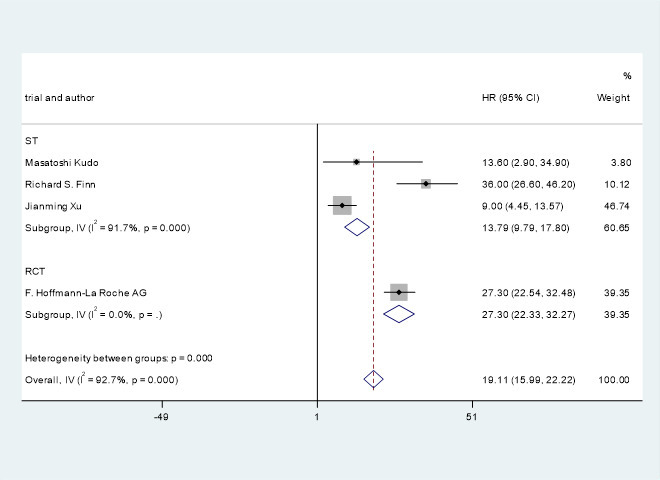
Pooled analysis of ORR in patients with HCC treated with immune checkpoint inhibitors combined with antiangiogenic drugs.

**Figure 7:**
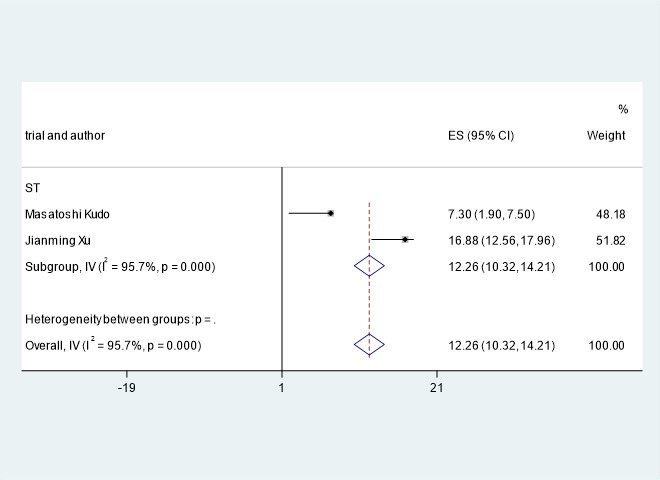
Pooled analysis of DOR in patients with HCC treated with immune checkpoint inhibitors combined with antiangiogenic drugs.

A pooled analysis of ORR and DOR in advanced/unresectable/metastatic HCC patients treated with immune checkpoint inhibitors combined with antiangiogenic drugs is shown in **Figures 6**, **7**.

### Adverse events

Adverse events of grade 1-2 common to ICIs in combination with antiangiogenic drugs in the treatment of hepatocellular carcinoma are shown in **Table 2**, including hypertension (26.8%, 95% CI=15.3%, 38.2%), diarrhea (23.6%, 95% CI=15.3%, 31.8%), fatigue (23.8%, 95% CI=19.3%, 28.3%), decreased appetite (22.8%, 95% CI=14.4%, 31.2%), hypothyroidism (15.1%, 95% CI=9.7%, 20.4%), rash (14.5%, 95% CI=9.2%, 19.8%). Common adverse reactions of immune checkpoint inhibitors combined with antiangiogenic drugs in the treatment of HCC are shown in **Table 2**.

**Table 2:** The common adverse events of immune checkpoint inhibitors combined with antiangiogenic drugs in the treatment of HCC.

Adverse events (Any grade)	ES	95% CI	Model	I^2^	P
Hypertension	26.8%	15.3%, 38.2%	Random-effects	95.2%	0.000
Diarrhea	23.6%	15.3%, 31.8%	Random-effects	89.0%	0.000
Fatigue	23.8%	19.3%, 28.3%	Fixed-effects	32.2%	0.000
Decreased appetite	22.8%	14.4%, 31.2%	Random-effects	90.4%	0.000
Hypothyroidism	15.1%	9.7%, 20.4%	Random-effects	72.2%	0.006
Rash	14.5%	9.2%, 19.8%	Random-effects	80.1%	0.000

**Figure 8:**
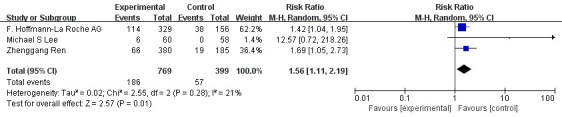
Forest plot of the incidence of hypertension (grade 1-2) in patients with HCC treated with ICIs and antiangiogenic drugs in combination or alone.

**Figure 9:**
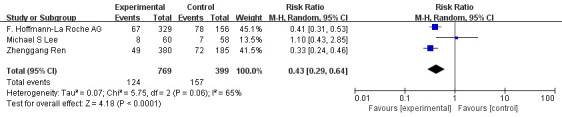
Forest plot of the incidence of diarrhea (grade 1-2) in patients with HCC treated with ICIs and antiangiogenic drugs in combination or alone.

**Figure 10:**
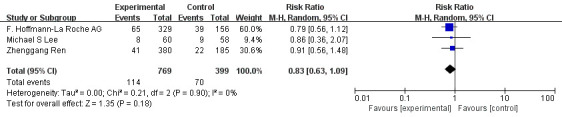
Forest plot of the incidence of decreased appetite (grade 1-2) in patients with HCC treated with ICIs and antiangiogenic drugs in combination or alone.

**Figure 11:**
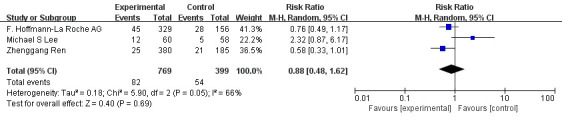
Forest plot of the incidence of rash (grade 1-2) in patients with HCC treated with ICIs and antiangiogenic drugs in combination or alone.

The 3 RCTs mentioned above were included in the meta-analysis. Meta-analysis indicated that compared with monotherapy, PD-1/PD-L1 inhibitor combined with antiangiogenic drugs had a higher incidence of grade 1-2 hypertension (OR=1.56, 95%CI 1.11-2.19, I^2^= 21%, P=0.01), as illustrated in **Figure 8**. In addition, the analysis results showed that compared with monotherapy, PD-1/PD-L1 inhibitor combined with anti-angiogenic drugs had lower incidences of diarrhea (OR=0.43, 95%CI 0.29-0.64, I^2^= 65%, P<0.0001), as illustrated in **Figure 9**. Moreover, there was no statistical difference in the incidence of decreased appetite and rash between the single drug and the combined drug, respectively (OR=0.83, 95%CI 0.63-1.09, I^2^= 0%, P=0.18), (OR=0.88, 95%CI 0.48-1.62, I^2^= 66%, P=0.69), as shown in **Figures 10**, **11**.

## Discussion

Hepatocellular carcinoma as one of the most common fatal tumors in the world, its fatality rate ranks the third in the world, the incidence ranks sixth, and is increasing year by year. The treatment of hepatocellular carcinoma needs to be based on the stage of the lesion, whether it is metastatic, the extent of metastasis, the patient’s physical condition, and their willingness to accept treatment methods. Targeted therapy for hepatocellular carcinoma first needs to identify the genetic mutation of this cancer. Based on mutations in the driver genes of hepatocellular carcinoma found by second-generation genetic testing, it is still unclear whether selective inhibition of these mutations can produce better clinical efficacy. For hepatocellular carcinoma, a major bottleneck is that there are no drugs that inhibit the most common genetic mutations in hepatocellular carcinoma, such as the TERT promoter, TP53, CTNNB1, AXIN1, ARID1A, or ARID2. However, the current clinical trial design of targeted therapy for hepatocellular carcinoma seldom considers genome-directed stratification, so this aspect needs urgent attention (28).

The existence of various types of immune cells in the liver establishes an immune microenvironment that strongly affects the occurrence and development of tumors. Using single-cell RNA sequencing, some studies have identified subsets of immune cells that may have distinct immune functions, and patients with certain subsets of features have significantly better outcomes. Therefore, the efficacy of immunotherapy is determined in part by the individual’s immune microenvironment. By specifically inhibiting PD-1, PD-L1 and CTLA-4, immunotherapy breaks the tumor immune tolerance mechanism and effectively delays tumor progression (11, 16). In recent years, as an important member of T lymphocyte immune deficiency related immunoglobulin superfamily costimulatory molecules, PD-1/PD-L1 contribute significantly to tumor immune escape. Anti-PD-1/PD-L1 therapy has become the most promising immunotherapy for hepatocellular carcinoma. The PD-1/PD-L1 inhibitors used in the studies included in this meta-analysis include avelumab, pembrolizumab, durvalumab, atezolizumab, SHR-1210 (anti-PD-1 antibody), and sintilimab. Among them, Avelumab, Atezolizumab, Durvalumab are humanized anti-PD-L1 IgG1 monoclonal antibodies. While Pembrolizumab and sintilimab are humanized anti-PD-1 IgG4 monoclonal antibodies. The efficacy and safety of PD-1/PD-L1 inhibitors from different sources alone and in combination in the treatment of hepatocellular carcinoma need to be further studied and discussed.

In the process of tumor growth and metastasis, angiogenesis is vital. Due to abnormal perfusion and increased permeability, newborn tumor vessels will lead to tissue hypoxia, lactic acid increase and necrosis, and then activate immunosuppression and inhibit the function of effector T cells. Antiangiogenic drugs can disrupt the vascular supply by blocking the VEGF/VEGFR signal pathway, the tumor is deficient in nutrients and oxygen. However, its effect on the overall survival rate of cancer patients is limited, and it rarely produces a lasting response (29). Additionally, crosstalk and VEGFR signaling downstream of the immune checkpoint axis may lead to synergistic effects of combination therapy on tumor cells (13). The antiangiogenic drugs used in the trials included in this Meta-analysis include axitinib, lenvatinib, ramucirumab, bevacizumab, apatinib, and bevacizumab biosimilar. Among them, axitinib, lenvatinib, and apatinib are small-molecule multi-target angiogenesis inhibitors, while ramucirumab and bevacizumab are macro-molecule single-target angiogenesis inhibitors. Axitinib is particularly specific for VEGFR, PDGFRβ and c-Kit. Lenvatinib inhibits angiogenesis by inhibiting the activity of VEGFR1, VEGFR2, VEGFR3, FGFR1-3, KIT, PDGFRα and RET. Apatinib targets include VEGFR1, VEGFR2, VEGFR3, PDGFR-β, C-KIT, FGFR1 and FLT3. Bevacizumab is a recombinant humanized monoclonal IgG antibody that can specifically bind to VEGF, block the binding of VEGF and its receptors, reduce angiogenesis, induce the degeneration of existing blood vessels, and thereby inhibit the tumor growth. Ramucirumab is a fully humanized IgG1 monoclonal antibody that selectively binds to the extracellular region of VEGFR2 and prevents the phosphorylation of VEGFR2. It is the only VEGFR2 monoclonal antibody that has been marketed in the world. Bevacizumab is an anti-vascular endothelial growth factor monoclonal antibody that specifically binds to VEGF-A and blocks angiogenic cell pathways. It is the world’s first approved anti-tumor angiogenesis targeted drug and the first recombinant humanized anti-VEGF monoclonal antibody. Different targets of the above anti-angiogenic drugs may exert different therapeutic effects, which also requires in-depth research in order to obtain the best treatment plan.

Both immunotherapeutic drugs and antiangiogenic drugs act on tumor microenvironment, and they have synergistic effect in theory. The mechanism of immune combined antiangiogenic therapy may include the following four aspects: a) antiangiogenic drugs reduce the activity of myelogenous suppressor cells and regulatory T cells and reshape the tumor microenvironment; b) antiangiogenic drugs block the VEGF-mediated inhibition of dendritic cell maturation, which makes T cells binding to tumor antigens start and activate more effectively; c) antiangiogenic drugs normalize tumor vascular structure and promote T cell infiltration into the tumor; d) antiangiogenic drugs restore anti-tumor immune function by killing tumor cells mediated by T cells. The combination of PD-1/PD-L1 and VEGF antibodies works well not only because of their additive effect on tumor growth inhibition, but also because they reprogram the immunosuppressive microenvironment to immunostimulatory microenvironment (30). In addition, studies have found that antiangiogenic drugs can induce the formation of high endothelial venules (HEV), and HEV is generally considered to be involved in lymphocyte homing. Therefore, researchers speculate that intratumor HEV will similarly promote T cell infiltration of tumors, tumor-associated high endothelial venules (TA-HEVs) are the main pathway for lymphocytes to enter tumors (31–33). In summary, immune checkpoint inhibitors combined with anti-angiogenic drugs have a synergistic mechanism in tumor treatment.The combined use of antiangiogenic drugs and immune checkpoint inhibitors still has some challenges to be solved. First, the normalization of tumor blood vessels induced by anti-tumor angiogenesis has a window period, and how to define the window period is still inconclusive. Second, how to optimize the dosage and administration frequency of anti-angiogenic drugs in combination therapy to avoid excessive inhibition of angiogenesis and bring maximum survival benefit to patients. In addition, although the expression level of PD-1/PD-L1, tumor mutation load, etc. can screen for dominant patients to a certain extent, more evidence shows that other components of the tumor microenvironment also play a role in determining the effectiveness of tumor immunotherapy. However, there are currently no biomarkers to guide the use of anti-angiogenic drugs, so combination therapy requires a more systematic evaluation method to pinpoint the benefit population.

This study shows that ICIs combined with antiangiogenic drugs can potentially improve OS and PFS in patients with advanced/unresectable/metastatic HCC. The OS and PFS of the patients treated with immune checkpoint inhibitors combined with antiangiogenic drugs were HR=15.84, 95% CI=15.39, 16.28, I^2^= 68.3%, p=0.043 and HR=5.93, 95% CI=5.41, 6.45, I^2^= 76.3%, p=0.000, respectively. Studies by Michael S Lee et al. (24) have shown that immune checkpoint inhibitors combined with antiangiogenic drugs can improve PFS from 3.4 months to 5.6 months. F.Hoffmann-La Roche Ltd (22) studies have indicated that immune checkpoint inhibitors combined with antiangiogenic drugs can improve PFS from 4.27 months to 6.83 months. The study by Zhenggang Ren et al. (23) showed that the combination of ICIs with antiangiogenic drugs improve PFS from 2.8 months to 4.6 months. The study by F. Hoffmann-La Roche Ltd (22) showed that the combination of immune checkpoint inhibitors with antiangiogenic drugs can improve OS from 13.40 months to 19.22 months.

The ORR and DOR of the patients treated with immune checkpoint inhibitors combined with antiangiogenic drugs were HR=19.11, 95% CI=15.99, 22.22, I^2^= 92.7%, p=0.000 and HR=12.26, 95% CI=10.32, 14.21, I^2^= 95.7%, p=0.000, respectively. The study by F. Hoffmann-La Roche Ltd et al (22) showed that the combination of immune checkpoint inhibitors with antiangiogenic drugs improved ORR from 11.9 months to 27.3 months.

The incidence of adverse events (grade 1-2) in patients with advanced/unresectable/metastatic HCC treated with immune checkpoint inhibitors combined with antiangiogenic drugs included hypertension (26.8%), diarrhea (23.6%), fatigue (23.8%), decreased appetite (22.8%), hypothyroidism (15.1%) and rash (14.5%). At the same time, the analysis results of RCTs may indicate that the combination therapy can reduce the incidence of diarrhea to a certain extent, and the incidence of decreased appetite and rash is not significantly different from that of single therapy. For other adverse reactions of combination therapy, clinicians and pharmacists can reduce the impact on patients’ medication compliance through medication monitoring and timely treatment. Therefore the above data reflects the tolerable safety of the combination of the two. This is presumably due to the normalization of blood vessels by antiangiogenic drugs, which improves the delivery of therapeutic drugs to the tumor, thereby reducing the dose of ICIs and reducing the risk of immune-related adverse effects.

Based on the funnel plot and Egger’s publication bias test, it is evident that the included article is not biased by publication.

The limitations of this study include: (1) there are few relevant randomized controlled trials; (2) the treatment cycles of the trials included are different; (3) the regimens of ICIs combined with antiangiogenic drugs are not uniform; (4) some of the I^2^ values in this meta-analysis were large, implying heterogeneity between studies; (5) subgroup analyses were not included in this meta-analysis; (6) there is currently a lack of sensitive and effective biomarkers for predicting antiangiogenic drugs combined with ICIs, hindering the adjustment of regimens in certain conditions. After overcoming these problems, the efficacy and safety of immune checkpoint inhibitors combined with antiangiogenic drugs in the treatment of advanced/unresectable/metastatic hepatocellular carcinoma may be clearer.

## Conclusion

To sum up, in the treatment of advanced/unresectable/metastatic hepatocellular carcinoma, the combination of immune checkpoint inhibitors and antiangiogenic drugs achieved better survival benefits than single use. In addition, the combination therapy has tolerable safety. This meta-analysis involves advanced/unresectable/metastatic hepatocellular carcinoma patients, thus providing new treatment options for patients with advanced HCC and new hope for the treatment prospects in this field. However, more RCTs are needed for further research. And the timing or sequence of each drug in combination and optimal regimens is unclear, nor is the optimal dose of each drug. In the future, basic research on the mechanism of the positive feedback loop between ICIs and antiangiogenic drugs should be increased and strengthened to help develop new prescriptions and design clinical studies. At the same time, biomarkers should be tested concurrently in more clinical trials. With the increase of effective evidence, the clinic can better decide the timing and sequence of administration of the combination to improve the efficacy and reduce the toxic and side effects.

## Data availability statement

The original contributions presented in the study are included in the article/supplementary material. Further inquiries can be directed to the corresponding author.

## Author contributions

The corresponding author SL is responsible for proposing research directions and innovations, the first author YZ completes the main research work and the writing of the paper, and the second and third authors assist in literature retrieval and article revision. All authors contributed to the article and approved the submitted version.

## Conflict of interest

The authors declare that the research was conducted in the absence of any commercial or financial relationships that could be construed as a potential conflict of interest.

## Publisher’s note

All claims expressed in this article are solely those of the authors and do not necessarily represent those of their affiliated organizations, or those of the publisher, the editors and the reviewers. Any product that may be evaluated in this article, or claim that may be made by its manufacturer, is not guaranteed or endorsed by the publisher.
